# Prevalence of anemia in renal insufficiency among HIV infected patients initiating ART at a hospital in Northeast Ethiopia

**DOI:** 10.1186/s12878-017-0071-2

**Published:** 2017-01-17

**Authors:** Temesgen Fiseha, Zemenu Tamir, Abdurahaman Seid, Wondmagegn Demsiss

**Affiliations:** Department of Clinical Laboratory Science, College of Medicine & Health Sciences, Wollo University, Dessie, Ethiopia

**Keywords:** HIV, ART initiation, Anemia, Renal insufficiency

## Abstract

**Background:**

Anemia is a strong predictor of mortality and poor quality of life among persons with either renal impairment or HIV infection. In this study, we investigated the prevalence of anemia and its association with renal insufficiency among HIV infected patients initiating ART at a hospital in Northeast Ethiopia.

**Methods:**

In this retrospective cohort study, records of 373 patients on ART were selected in Dessie Referral hospital, South Wollo, Northeast Ethiopia from September 2010 to August 2013. Socio-demographic and clinical characteristics of the study patients were collected using standardized data extraction instrument. The abbreviated 4-variable Modification of Diet in Renal Disease (MDRD) study equation was used to estimate renal function (GFR) from serum creatinine. SPSS version 20.0 statistical software was used for data analysis.

**Results:**

The prevalence of anemia at the time of ART initiation was 34.4%; with 20.5, 12.3 and 1.6% mild, moderate and severe anemia, respectively. Renal insufficiency was present in 27.9% of patients and was associated with a high prevalence of anemia (74%). The prevalence of anemia increased with stage of insufficiency, from 23.7% in stage 1 to 100% in stage 4. Impaired renal function (eGFR < 60 mL/min/1.73 m^2^) was associated with a higher risk of all forms of anemia; i.e., mild (AOR = 3.96; 95% CI: 2.76–5.69), moderate (AOR = 2.21; 95% CI: 1.16–4.19) and severe anemia (AOR = 5.89; 95% CI: 1.02–12.03).

**Conclusion:**

HIV infected patients with renal insufficiency had a higher prevalence of anemia compared to patients with normal renal function. Thus, screening of these patients for anemia and renal insufficiency at base line should be critical not only to reduce mortality but also to improve clinical outcomes.

## Background

Anemia is recognized to be the most common hematological abnormalities in patients with human immunodeficiency virus (HIV) infection and is an important clinical problem in these patients both before and after the advent of combination antiretroviral therapy (ART) [1, 2]. Evidences indicate that anemia among these patients was associated with a much faster rate of disease progression and decreased quality of life, and was a strong prognostic marker for death [3–5]. Depending on the study setting, anemia can be found in 63–95% of those with HIV infection at some point during the course of their disease which might be caused by a wide range of etiologic factors leading to decreased red blood cell (RBC) production, increased RBC destruction, or ineffective RBC production [6, 7]. Patients with more advanced HIV disease or a lower CD4 cell count had higher rates of anemia [8].

As in HIV, anemia occurs commonly in renal insufficiency, the result of decreased production of erythropoietin, a signaling molecule that stimulates red blood cell production. A decline in renal function appeared to constitute a significant risk of anemia, and the prevalence of anemia increases significantly with progressive renal impairment [9]. Among persons with renal insufficiency, anemia has been associated with substantial clinical and public health outcomes including increased risk of morbidity, mortality and poor quality of life [10]. As lower hemoglobin levels associate with major clinical outcomes at all levels of function, treatment of anemia ameliorates the loss of kidney function and progression to renal failure and improves quality of life and various health measures.

Anemia is a strong predictor of mortality and poor quality of life among persons with either renal impairment or HIV infection [3, 5, 10], and a study on the risk of anemia suggest that HIV infection and impaired renal function act synergistically to increase the risk for the development of anemia. According to this study, HIV infected persons with impaired renal function had 5–15 times greater risk of developing anemia than those with either risk factor alone, suggesting a higher risk of anemia associated morbidity and mortality [11]. Despite the fact that HIV infected persons with impaired renal function have a higher risk of developing anemia, and thus, suffer extra morbidity and mortality than those without renal impairment; relatively little is known about its prevalence among HIV infected adults in Ethiopia. In this study, we assessed the prevalence of anemia and its association with renal insufficiency among HIV infected adults at the time of ART initiation in Dessie referral hospital of Southern Wollo, Northeast Ethiopia.

## Methods

### Study setting and study population

A retrospective study was conducted by reviewing hospital data of patients who were receiving HAART at the HIV clinic of Dessie Referral Hospital (DRH) in Southern Wollo, Northeast Ethiopia from September 2010 to August 2013. The hospital serves as a referral center for the surrounding zones and provides HIV/AIDS interventions including free diagnosis, treatment and monitoring. The Hospital launched Antiretroviral Therapy (ART) in September 2003, and 5,245 were on ART and 502 were on pre-ART during data collection. Among those, a total of 373 (140 males and 233 females) HIV infected individuals data at the time of ART initiation were randomly selected for this study. The age range of the patients was above 18 years. Patients who had incomplete recording at baseline and those who were pregnant at the time of ART initiation were excluded from this study. Patients with baseline evidence of hospitalization, acute illnesses (fever) and dialysis therapy were also excluded.

Before collection of the data, a data extraction tool was prepared and pre tested. Additionally, the nurses who were selected to collect the data were given one day training on the data extraction tool and how to collect data from registers to ensure no selection or information bias was introduced at the time of data collection. During data collection, the principal investigator supervises the data collectors and examined the tools for accuracy and completeness.

### Laboratory measurements and definitions

Hemoglobin (Hgb) values were determined using the hematology analyzer Cell-Dyn 1800 (Abbott Laboratories Diagnostics Division, USA) and CD4+ T cells were assayed using the BD FACSCOUNT system (Becton Dickenson and Company, California, USA). Anemia was defined according to the WHO criteria: Hgb concentration <13 g/dl for males and < 12 g/dl for females. We classified anemia as mild (11–12.9 for males and 11–11.9 g/dl for females) moderate (8–11 g/dl) and sever (<8 g/dl). Biochemical analyses were done on HumaStar 80 clinical chemistry analyzer (Human Diagnostics, Germany) using kits supplied by the manufacturer. Serum creatinine concentration was assayed by a kinetic Jaffe’ method as mg/dl with calibration traceable to IDMS reference material NIST SRM 909B level 2. Kidney function (eGFR) was assessed according to the simplified version of the Modification of Diet in Renal Disease (MDRD) study equation [12]: 186 × SCr(mg/dl)^-1.154^ × age(years)^-0.203^ × 0.742 (if female) × 1.210 (as our population are Africans).

Staging of renal function was based on the National Kidney Foundation Disease Outcomes Quality Initiative (K/DOQI) classification [13]. Stage 1 renal impairment was defined as normal or increased eGFR (eGFR ≥ 90 ml/min/1.73 m^2^). Mild, moderate, and severe renal impairment were defined as eGFR 60–89.9, 30–59.9 and 15–29.9 ml/min/1.73 m^2^, respectively. For the purposes of this study, impaired renal function was defined as eGFR <60 ml/min/1.73 m^2^.

### Statistical analysis

The data was entered in to “EpiInfo version 3.1” and was exported to SPSS version 20.0 statistical software for analysis Normally distributed and continuous variables were expressed as mean ± standard deviation (SD), and non-normally distributed variables were presented as medians (quartiles 25 and 75%). Chi-square (×2) test was used to compare proportions. Multivariate logistic regression was used to calculate adjusted odds ratios (OR) and the corresponding 95% confidence intervals (CI). *P* value < 0.05 was used to indicate statistical significance.

### Ethical consideration

Study protocol was approved by the Institutional Review Board of College of Medicine and Health Sciences of Wollo University. Official letter of cooperation was written to Dessie referral hospital, and permission from the ART clinic of the hospital, where the data collection took place was obtained. Confidentiality of the patient’s information was strict and no information was handed over to a third party.

## Results

### Clinical characteristics of the study participants

A total of 373 HIV positive individuals (140 males and 233 females) were assessed for the presence of anemia and renal impairment at the time of ART initiation at the ART clinic of Dessie referral hospital. The mean ± SD age of the study participants was 34.6 ± 10.8 years, ranging from 18 to 76 years and 89.3% of them were below 50 years old. The median CD4 count was 182.0 cells/mm^3^ (IQR: 94–313), and 80.4% had CD4 < 350 cells/mm^3^. Only, 97 (26%) of study participants had a positive history of opportunistic infections (OI), and 147 (39.4%) and 135 (36.2%) were classified as WHO stage III/IV. Mean haemoglobin (Hgb) level was 12.84 ± 2.12 g/dl. Mean serum creatinine (SCr) level was 0.94 ± 0.43 mg/dl. The mean eGFR value was 115.91 ± 43.45 ml/min/1.73 m^2^ (Table 1).

**Table 1:** Demographic and clinical characteristics of HIV infected adults at the time of ART initiation in DRH of Southern Wollo, South Eastern Ethiopia, 2015 (*n* = 372)

Characteristics		
Age (year), mean ± SD		34.57 ± 10.8
Age group, n (%)	18–29 Years	131 (35.1)
	30–39 Years	131 (35.1)
	40–49 Years	71 (19.0)
	>50 Years	40 (10.7)
Sex, n (%)	Male	140 (37.5)
	Female	233 (62.5)
Opportunistic infections, n (%)	Yes	97 (26.0)
	No	276 (74.0)
WHO clinical stage, n (%)	Stage I/II	226 (60.6)
	Stage III/IV	147 (39.4)
CD4 count (Cells/mm^3^), median (IQR)		182 (94–313)
CD4 count (Cells/mm^3^), n (%)	<200	206 (55.2)
	200–350	94 (25.2)
	>350	73 (19.6)
Haemoglobin (Hgb), mean ± SD		12.84 ± 2.12
Serum Creatinine (SCr), mean ± SD		0.94 ± 0.43
eGFR, mean ± SD		115.9 ± 43.45

### Prevalence of anemia

Anemia was present in 34.4% of the study participants, with 20.5, 12.3 and 1.6% described as mild, moderate and severe anemia respectively. The overall prevalence of anemia was 41.4% among males and 34.3% among females, and gender had no a significant influence on the overall prevalence of anemia at the time of ART initiation in our study participants (*P* = 0.169). The overall prevalence of anemia was 29.1, 36.2, 34.6 and 55.9% among patients whose age group was within 18–29, 30–39, 40–49 and >50 years, respectively, and was significantly higher among participants > 50 years old than ≤ 50 years old: 70.0% vs. 34.1% *(P* = 0.004). The overall prevalence of anemia significantly increased with WHO stage of HIV disease, ranging from 19.1% in Stage 1 to 63.6% in Stage 4 *(P* = 0.007) (Table 2).

**Table 2:** Overall prevalence of Anemia among HIV infected adults at the time of ART initiation in DRH of Southern Wollo, South Eastern Ethiopia, 2015 (*n* = 372)

Variables		N (%)	*P*-value
Age			
	≤ 50 years	117 (34.1)	< 0.001
	> 50 years	21 (70.0)	
Sex			
	Male	58 (41.4)	0.169
	Female	80 (34.3)	
History of opportunistic infections			
	Yes	35 (36.1)	0.800
	No	103 (37.3)	
WHO clinical stage			
	Stage I/II	66 (31.1)	0.007
	Stage III/IV	72 (44.7)	
CD4 count			
	< 200 cells/mm^3^	100 (48.5)	< 0.001
	200–350 cell/mm^3^		
	> 350 cells/mm^3^	9 (12.3)	
Serum creatinine			
	> 1.5 mg/dl	36 (94.7)	< 0.001
	≤ 1.5 mg/dl	102 (30.4)	

Based on baseline CD4 count, there was a significant difference in the prevalence of anemia within the three CD4 categories. The prevalence of overall anemia increased with decreasing CD4 count, with 48.5% among patients with CD4 count < 200 cells/mm^3^, and 30.9 and 12.3% among those with CD4 count of 200–350 and >350 cells/mm^3^ respectively (*P* < 0.001). However, there were no significant differences in anemia prevalence among participants with and without a history of opportunistic infection (*P* = 0.800). Abnormal serum creatinine (SCr), defined as SCr levels > 1.5 mg/dl, was present in 6.1% of participants and was associated with a higher prevalence of anemia than its absence: 95.2% vs. 31.3% (*P* < 0.001) (Table 2).

Renal insufficiency, defined as an estimated GFR (eGFR) less than 90 mL/min/1.73 m^2^, was present in 104 (27.9%) of participants at the time of ART initiation. Of these, 64 (17.2%), 38 (10.2%) and 2 (0.5%) of them were mild, moderate and severe renal insufficiency with eGFR of 60–89.9, 30–59.9 and 15–29.9 mL/min/1.73 m^2^, respectively. The prevalence of anemia was 74% in people with renal insufficiency (Table 3). The prevalence of anemia in people without renal insufficiency was 22.7%. The prevalence of overall anemia among participants with impaired renal function (eGFR < 60 mL/min/1.73 m^2^) was significantly higher (94.3%) than those without impaired renal function (28.5%, *P* < 0.001).

**Table 3:** Prevalence of anemia associated with worsening renal insufficiency (*n* = 372)

Stage (eGFR, ml/min/1.73 m^2^)	Description	N (%)	Anemia N (% total)
1 (>90)	Normal or high GFR	269 (72.1)	61 (74.0)
2 (60–89.9)	Mild renal insufficiency	64 (17.2)	38 (60.3)
3 (30–59.9)	Moderate renal insufficiency	38 (10.2)	36 (94.7)
4 (15–29.9)	Severe renal insufficiency	2 (0.5)	2 (100)

The prevalence of anemia among HIV- infected patients with renal insufficiency was significantly higher compared to patients with normal renal function (*P* < 0.001). The prevalence of anemia increased with stage of renal insufficiency, ranging from approximately 23.7% in Stage 1 to 100% in Stage 4. The prevalence of overall anemia, and of mild anemia increase significantly with stage of renal insufficiency (both *P* < 0.001), but not the prevalences of moderate and severe anemia (*P* = 0.080 and *P* = 0.115). Impaired renal function, defined as eGFR < 60 mL/min/1.73 m^2^, was associated with an increased risk of overall anemia with an odds ratio of 3.311 (2.725–4.022, *P* < 0.001). Impaired renal function contribute to the risk of all forms of anemia with odds ratios of 3.964 (2.761–5.691, *P* < 0.001), 2.207 (1.162–4.192, *P =* 0.019) and 5.886 (1.018–12.030, *P* = 0.026) for mild, moderate and severe anemia, respectively.

## Discussion

Anemia is a frequent complication of HIV infection and can have serious implications, varying from functional and quality of life decrements to an association with disease progression and decreased survival [14]. As recovery from anemia increase survival, screening for anemia among HIV positive population at base line should be aggressive and critical not only to reduce mortality, but also in suggesting ways to improve quality of life [2, 8]. In this study, we investigated the prevalence of anemia and its association with impaired renal function among a population of HIV-infected patients at the time of ART initation.

Based on the results of this study, more than 37% of HIV infected patients initiating ART had anemia, with 23.1, 12.3 and 1.6% mild, moderate and severe anemia, respectively. Our prevalence estimate of anemia was lower than those reported in studies conducted in other parts of the country; 42.3, 42.9 and 86.5% in North West Ethiopia, Addis Ababa and South Ethiopia, respectively [15–17]. This is also lower than estimate of 70.5% prevalence of anemia among HIV-infected patients at treatment initiation but higher than 25.8% estimates in Southern Africa [18, 19]. These differences in anaemia prevalence might be due to the heterogeneity of study population and differences in the study settings in terms of nutritional status and stages of HIV infection. Furthermore, despite the fact that a WHO definition for anemia was proposed more than 45 years ago [20], comparative studies of HIV-related anemia have used non-standardized definitions, varying prevalence estimates between settings and biasing the scope of the problem.

Reduced renal function is an important complication of HIV infection associated with a significant risk of CVD, heart failure and faster disease progression [21, 22]. In this study, 104 (27.9%) of HIV infected patients had renal insufficiency. This finding is nearly similar to that from a study done in Zambia where renal insufficiency was found in 33.5% of the HIV infected patients initiating ART [23], but lower than 36.9% reported in the Uganda study [24] and 38 and 39% reported in two Nigeria studies [25, 26] who used the same cut off of eGFR <90 mL/min/1.73 m^2^ to define renal insufficiency. However, the Uganda study used Chronic Kidney Disease Epidemiology Collaboration (CKD-EPI) and the Zambian and Nigerian studies used Cockroft and Gault (CG) formula for calculating eGFR. When renal impairment was defined as eGFR < 60 mL/min/1.73 m^2^, 10.7% of our HIV infected participants had impaired renal function. Our finding is lower than the recent finding of 24% in Nigeria [26] and higher than findings in Ghanaian HIV infected HAART-naïve patients that reported 9.1% using the same definition with MDRD formula [27].

As decreased renal function at baseline may identify HIV infected patients at increased risk of mortality and kidney disease progression [28], regular screening of these population for renal impairment at base line should be critical not only to reduce mortality but also to improve patient outcomes. One of the many known complications of decreased renal function is anemia, a condition in which the number of circulating red blood cells, and therefore the level of hemoglobin, is lower than normal. Anemia is a strong predictor of mortality and poor quality of life among persons with either renal impairment or HIV infection [3, 5, 10], and HIV-infected persons with renal impairment had a greater risk of developing anemia than patients with either risk factor alone [11]. The additional risk of anemia among HIV-infected patients with renal impairment when compared to HIV-infected patients with normal renal function suggests a higher risk of anemia-associated morbidity and mortality.

In the present study we found that HIV- infected patients with renal insufficiency have a higher prevalence of anemia (74%) compared to patients with normal renal function (74% vs. 22.7%, *P* < 0.001). The results of the above study suggest that HIV infection and impaired renal function interact to exacerbate hemoglobin decline, even among persons receiving effective HIV treatment [11]. The prevalence of anemia increased with stage of renal impairment, from 23.7% at stage 1 to 100% at stage 4. HIV- infected patients with impaired renal function had a risk of having mild, moderate and severe anemia that was 3.9, 2.2 and 5.8 times greater than those without impaired renal function. Dickson and co-authors [29] recently reported that anemia increases in prevalence with severity of renal disease, and anemia in either renal insufficiency or HIV patients is associated with increased morbidity and mortality; therefore, a prompt diagnosis of anemia is warranted, as it may impact clinical treatments and outcomes in this population. Moreover, as recovery from anemia increase survival, screening for anemia among HIV positive population with or without renal impairment at base line should be aggressive and critical not only to reduce mortality, but also in suggesting ways to improve quality of life.

## Conclusion

In conclusion, the prevalence of anemia and renal insufficiency was higher in this HIV infected adult population at the time of ART initiation. HIV infected patients with renal insufficiency had a higher prevalence of anemia compared to patients with normal renal function. Thus, screening of these patients for anemia at base line should be critical not only to reduce mortality but also to improve clinical outcomes.
